# Does intraoperative anesthesia handovers associated with adverse outcomes? A systematic review and meta-analysis

**DOI:** 10.3389/fmed.2026.1815524

**Published:** 2026-06-11

**Authors:** Congcong Zou, Hongyang Chen, Weiyi Zhang

**Affiliations:** 1 Department of Anesthesiology, West China Hospital, Sichuan University, Chengdu, China; 2 Anesthesia and Surgery Center of West China Xiamen Hospital, Sichuan University, Xiamen, China; 3 Department of Anesthesiology, Xizang Hospital of West China Hospital, Sichuan University, Lhasa, China

**Keywords:** adverse outcomes, anesthesia, intraoperative anesthesia handover, morbidity, mortality, operating rooms, surgery

## Abstract

**Background:**

Intraoperative anesthesia handover is a common occurrence; however, limited research has explored its impact on patient outcomes. The purpose of this systematic review and meta-analysis was to assess the effects of anesthesia handovers on adverse outcomes in surgical settings.

**Methods:**

All clinical studies that specifically investigated the association between anesthesia handovers and adverse patient outcomes were included. The MEDLINE, Cochrane Library trials, PubMed, Embase, and Web of Science databases were searched from inception to June 15, 2025. The risk of bias was assessed using the Newcastle-Ottawa Scale (NOS). Using STATA and R statistical software, exploratory and cumulative meta-analyses were conducted using a random-effects model for adjusted ratio risks (aRR) with 95% confidence intervals [95% CI]. Subgroup and sensitivity analyses were also performed given potential heterogeneity.

**Results:**

Meta-analyses of 14 studies revealed a significant association between anesthesia handovers and several adverse outcomes, including composite in-hospital mortality and morbidity (aRR = 1.44, 95% CI 1.23–1.69, I^2^ = 97.6%), in-hospital mortality (aRR = 1.49, 95% CI 1.15–1.91, I^2^ = 95.5%), morbidity (aRR = 1.35, 95% CI 1.18–1.54, I^2^ = 98.0%), length of intensive care unit (ICU) stay (aRR = 1.33, 95% CI 1.23–1.44, I^2^ = 82.3%), and the number of emergency department visits within 90 days of index surgery (aRR = 1.08, 95% CI 1.06–1.11, I^2^ = 0.0%), whereas only a non-significant association was observed for readmission within 30 days (aRR = 1.09, 95% CI 0.97–1.22, I^2^ = 91.9%). Furthermore, subgroup analyses indicated that the association between anesthesia handover and adverse outcomes was not modified by either surgical severity or a specific level of anesthesia handover.

**Conclusions:**

Intraoperative anesthesia handovers are generally associated with an increased risk of mortality and morbidity in surgical patients. Future research should prioritize identifying the specific characteristics of anesthesia handovers that affect patient safety.

**Systematic review registration:**

https://www.crd.york.ac.uk/prospero/, identifier: CRD420251140062.

## Introduction

More than 234 million patients undergo surgery annually worldwide (1). Surgical patients are at a high risk for inadequate handovers due to their high frequency, informal patterns, breakdowns of care continuity, and prone to information omission or misinterpreted transitions throughout the whole phase of perioperative care (2). Anesthesia care has been reported to be the major root cause of anesthesia-related sentinel events in the perioperative environment (3). The potential adverse outcomes ranged from minor delays to treatment, severe disability, critical morbidity, and even mortality (4, 5). As a common practice in the perioperative setting, handover encompassed two core meanings: duty relief among anesthesia providers of equivalent training and responsibilities; the postoperative transition of care from the operating room (OR) to the post-anesthesia care unit (PACU) (6, 7).

In this systematic review, we mainly focused on anesthetic transition that occurred only in the OR. In addition, we focused on “shift-to-shift” handoff; in other words, it refers to duty relief (not breaks or short-term transfer of patient care with planned transfer back to the original care provider) by providers with competent training and responsibilities (8). Anesthesia handover is defined as perioperative transitions among anesthetic providers; in other words, any health worker providing anesthetic care irrespective of the level of training or supervision (9). It includes handovers among attending anesthesiologists and medical-directed anesthesia providers, including residents and fellows, certified registered nurse anesthetists (CRNAs), and student nurse anesthetists (10). A recent randomized controlled trial published in the *Journal of the American Medical Association (JAMA)* demonstrated that intraoperative anesthesia handovers were not associated with an increased risk of adverse patient outcomes (11). This result contradicts those of two prior systematic reviews, which indicated that patients undergoing intraoperative anesthesia handovers had a higher risk of adverse outcomes than those in the no-handover group (4, 5).

Currently, there is a lack of universally accepted standard operating procedures, clinical guidelines, or consensus statements for handover details among anesthesia providers. Additionally, uncertainty persists regarding the impact of intraoperative anesthesia handovers on adverse outcomes in surgical patients, with the potential for emerging studies to alter the current body of evidence on this topic. Additional studies may have changed the results of the present study. Therefore, the goal of this systematic review was to synthesize the latest available evidence regarding the association between intraoperative anesthesia handovers and adverse outcomes in surgical patients.

## Methods

This systematic review followed the Preferred Reporting Items for Systematic Reviews and Meta-Analyses (PRISMA). The protocol was registered in PROSPERO (CRD420251140062). The West China Xia Men Hospital Human Research Ethics Board waived the need for informed consent for this systematic review and meta-analysis. No new human subjects were recruited; therefore, no additional patient consent was required.

### Eligibility criteria

We included all prospective or retrospective clinical studies that quantitatively investigated the association between intraoperative anesthesia handovers and surgical patient morbidity or mortality outcomes. Specifically, the handover was performed between anesthesia providers during a surgical and anesthetic procedure; in other words, providing intraoperative relief or transferring patient care to an incoming anesthesia provider. An anesthesia provider was defined as any healthcare professional with specific training in anesthesia such as physician anesthetists, postgraduate trainees (residents and fellows), nurse anesthetists, or anesthesia assistants. Studies could include patients of any age who underwent any type of surgical procedure that required anesthesia. Language restrictions were not included in this study.

### Exclusion criteria

Handovers other than those in the operating room were excluded, such as handovers in the ward, PACU, or intensive care unit. Letters, commentaries, editorials, opinion pieces, and abstracts were excluded from analysis.

### Search strategy

The search strategies (Supplementary Appendix 1) were designed by two reviewers (CCZ and HYC) and reviewed by a third reviewer (WYZ). The MEDLINE, Embase, PubMed, Cochrane Library, and Web of Science electronic databases were searched from their inception until June 15, 2025. Reference lists from the included studies and relevant systematic reviews and registration centers, such as ClinicalTrials.gov were also searched. Search terms included both MeSH terms (“patient handoff,” “patient safety”) and entry terms (“patient handover,” “patient sign out,” “anesthesia handoff,” “anesthesia handover,” “surgical procedures,” “intraoperative care,” “composite adverse events”).

### Study selection

Two independent reviewers (CCZ, HYC) screened articles for inclusion in duplicate using a web-based systematic review software—Covidence (covidence.org). Consensus or consultation with a third reviewer (WYZ) was used to resolve any disagreement. Titles and abstracts were assessed for eligibility, followed by full texts of those determined to be eligible.

### Data extraction

According to the guidance of the Cochrance Handbook (12), the Two independent reviewers collected the data using a customized data extraction form according to the Cochrane Handbook guidelines (12). For this review, the following information was retrieved and tabulated for all selected studies: publication details (e.g., first author's name, publication date, and country of data collection), study design (e.g., Observational vs. interventional, respective vs. prospective, method for assessing handovers, sample size), inclusion criteria, exclusion criteria, participant demographic data, and patient outcome measures (e.g., primary outcome such as ratio risk of composite in-hospital mortality and morbidity, secondary outcomes such as ratio risk of in-hospital mortality, morbidity, intensive care unit stay, readmission within 30 days, the number of emergency department visits within 90 days of the index surgery etc.), and clinical contest (e.g., type of surgery, elective vs. crisis situation) and intervention details (e.g., Handover strategy/tool names and types [e.g., electronic reminder, checklist, protocol], definition of handovers, implementation strategy), descriptive characteristics of handovers (e.g., frequency, error rate, and associations with other variables).

### Risk of bias

The risk of bias was determined using the Newcastle-Ottawa scale for included 13 cohort studies (Supplementary Appendix 2). Reviewers assessed the risk of bias independently and in duplicate using consensus or a third consultation to resolve disagreements (WYZ).

### Statistical analysis

We conducted a narrative synthesis of the results using specific qualitative and quantitative information from each study, as summarized in the tables. We also conducted a meta-analysis to quantify the effects of handovers on patient outcomes in a subset of comparable studies. The meta-analysis was conducted using a random-effects model with STATA version 18.0. MP software (STATA Corp., College Station, TX, USA). The effect estimates of dichotomous outcomes are presented as aRR with 95% CI. Statistical heterogeneity was assessed using the I^2^ statistic.

Subgroup analysis was performed to explore potential sources of heterogeneity. Leave-one-out sensitivity analysis was conducted to assess the stability and robustness of pooled effect estimates. Each study was sequentially omitted, and the overall effect size was recalculated iteratively. The conclusion was deemed reliable if the pooled effect and heterogeneity remained stable after excluding any single study; otherwise, the outlying study was recognized as the main source of heterogeneity. Cumulative meta-analysis was further performed with studies pooled incrementally in chronological order. This approach evaluated the temporal stability of results and identified individual studies that exerted undue influence on the overall meta-analytic findings. both of these sensitivity analysis were conducted using the meta package in R (The R Foundation; http://www.r-project.org; version 4.5.1).

## Results

### Study selection

**Figure 1:**
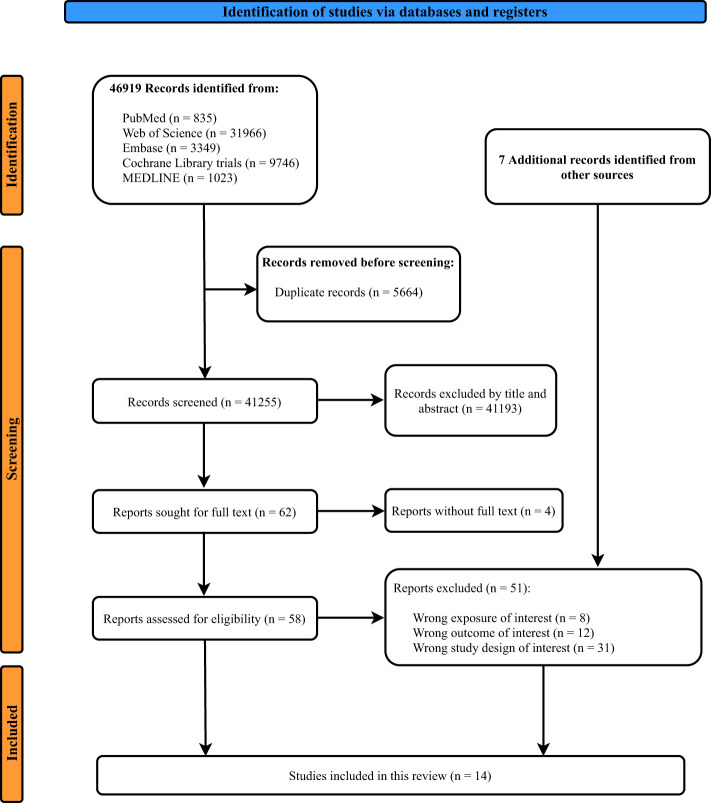
Flowchart of the included studies.

A total of 46,919 records were identified for subsequent screening. After excluding 5,664 duplicate records, 41,193 were deemed irrelevant, and four were excluded owing to the unavailability of full text. The remaining 58 studies were assessed for their eligibility. Concurrently, seven additional 7 records, identified from other sources were subjected to an eligibility assessment. Of the 65 records, 51 were excluded for the following reasons: inappropriate exposure of interest, mismatched outcomes of interest, and incorrect study design. Finally, 14 studies were included in this systematic review and meta-analysis (Figure 1).

### Characteristics of included studies

Table 1 presents the characteristics of the studies included. The 14 included studies published in the last two decades had a large sample size of 1,074,240. Of the 14 included studies, 13 were retrospective cohort studies (3, 10, 13–23), and the remaining one was an RCT (11). These studies were primarily conducted across four countries: the United States (3, 10, 14, 15, 17–19, 21, 22), Canada (13, 16, 20), Germany (11), and China (23).

**Table 1:** General characteristics of 14 included studies.

References	Study design	Time	Country	Surgery type	Sample size	Newcastle-Ottawa scale
Saager et al. (10)	Single-center retrospective cohort study	2005–2012	USA	Non-cardiac surgery	135,810	8
Hudson et al. (13)	Single-center retrospective cohort study	1999–2009	Canada	MAJOR cardiac surgery	14,421	8
Anastasian et al. (14)	Single-center retrospective cohort study	2008–2013	USA	Inpatient surgery	37,824	8
Hyder et al. (3)	Single-center retrospective cohort study	2006–2010	USA	Elective colorectal surgery	927	8
Terekhov et al. (15)	Single-center retrospective cohort study	2005–2014	USA	Inpatient surgery	140,754	8
Jones et al. (16)	Single-center retrospective cohort study	2009–2015	Canada	Major inpatient surgery	313,066	8
O'Reilly-Shah et al. (17)	Single-center retrospective cohort study	2014–2017	USA	Inpatient surgery	12,111	8
Hannan et al. (18)	Single-center retrospective cohort study	2010–2016	USA	Cardiac surgery	103,102	8
Kannampallil et al. (19)	Single-center retrospective cohort study	2013–2018	USA	Pediatric surgery	78,321	8
Meersch et al. (11)	Multiple-center RCT	2019–2021	Germany	Major inpatient surgery	1,772	NA
Sun et al. (20)	Single-center retrospective cohort study	2008–2019	Canada	Cardiac surgery	102,156	8
Bloom et al. (21)	Single-center retrospective cohort study	2016–2021	USA	Cardiac surgery	5,937	8
Saha et al. (22)	Single-center retrospective cohort study	2016–2021	USA	Non-cardiac surgery	121,077	8
Zhang et al. (23)	Single-center retrospective cohort study	2012–2020	China	Elective thoracic surgery	6,962	8

Regarding the type of surgery and handover incidence, the 14 included studies were categorized as follows: two studies (total sample size of 314,838 patients) focused on major inpatient surgery (11, 16), and the average anesthetic handover rate was 2.2%; four studies (total sample size of 225,616 patients) focused on major cardiac surgery (13, 18, 20, 21), with an anesthetic handover rate averaging 5.7%; one study (total sample size of 78,321 patients) focused on pediatric surgery (19), in which 6.9% of patients underwent anesthetic handover; and four studies (total sample size of 264,776 patients) focused on non-cardiac surgery (3, 10, 22, 23), with an anesthesia handover incidence of 38.1%. The handover rate in noncardiac surgeries was much higher than that in major cardiac surgeries.

Regarding the definition of anesthesia handover, each independent study defined it as one that occurred at least at the attending level. To mitigate the impact of potential confounding variables, most studies were statistically adjusted for well-established confounding factors affecting prevalence, including age, sex, race, American Society of Anesthesiologists (ASA) physical status classification, surgery duration, and surgery severity. In contrast, a subset of other studies further incorporated adjustments for multiple underlying chronic comorbidities to enhance the robustness of their analyses. The additional details are provided in Table 2.

**Table 2:** Specific characteristics of included studies.

References	Anesthesia handover (*n*, %)	Handover definition	Adjusted potential confounding variables	Primary outcome	Secondary outcome
Saager et al. (10)	One AH: 27,982, 21%; two AH: 15,102, 11%; three AH: 6172, 5%; four or more AH: 3,910, 3%	Handovers among attending anesthesiologists; handovers among medical-directed anesthesia providers (residents and fellows, CRNAs, and student nurse anesthetists)	Age, sex, race, ASA physical status, start time of surgery, duration of surgery, principal diagnosis and procedure, severity of procedure	Collapsed composite of in-hospital mortality/morbidity: OR 1.08 (1.05–1.10); attending AH OR 1.07 (1.03–1.12); residents or nurses AH OR 1.07 (1.04–1.11)	SERIOUS cardiac: OR 1.19 (1.13–1.25); respiratory: OR 1.06 (0.96–1.17); gastrointestinal: OR 1.18 (1.12–1.23); urinary: OR 1.06 (0.98–1.14); bleeding: OR 1.19 (1.13–1.26); infectious complications: OR 1.15 (1.09–1.22).
Hudson et al. (13)	One AH: 966, 6.7%	Transfer of patient care from one attending cardiac anesthesiologist to another during the intraoperative period	Age, sex, preoperative creatinine, EuroScore, aortic cross-clamp time, history of peripheral vascular disease, previous cardiac surgery, left ventricular class, operative priority, unstable angina, recent myocardial infarction, chronic obstructive pulmonary disease, active endocarditis, transfusions, return to CPB, reopening before leaving the operating room, surgery type, and atrial fibrillation	Match-adjusted in-hospital mortality: OR 1.425 (1.013–2.006); match-adjusted major morbidity: OR 1.274 (1.037–1.564)	Composite index of major postoperative morbidity [Periop MI OR 0.645 (0.378–1.101), CVA OR 2.132 (1.369–3.321); CVVH OR 1.250 (0.853–1.832); Vent >48hrs OR 1.354 (1.089–1.684)]
Anastasian et al. (14)	One AH: 8027, 21.2%	A case supervision transferred from one attending anesthesiologist to another	Age, gender, ASA physical status, obesity or not, difficult intubation or not, total case duration, estimated blood loss, total crystalloid administration, total colloid administration, total blood administration, day of surgery, emergency surgery, patient position during surgery, case end time of day	Delayed extubation at the end of the surgical case: RR 1.14 (1.03–1.25)	NA
Hyder et al. (3)	One AH: 287, 31.0%; two or more AH: 110, 11.9%	Handoff in care among attending anesthesiologists, the number of handoffs is equal or nearly equal to the number of anesthesiologists minus 1; in-room providers is consisted of anesthesiology residents and CRNAs; a high-provider team would include at least one handoff among attending anesthesiologists and at least 2 handoffs among in-room providers	Age, ASA classification, wound classification, work relative value units, operative duration, estimated blood loss	Major complication and/or death within 30 days of index surgery OR 1.44 (1.09–1.91); High–provider team OR 2.04 (1.15–3.61); the number of in-room providers OR 1.39 (1.01–1.92); all anesthesia providers OR 1.58 (1.20–2.08)	NA
Terekhov et al. (15)	One AH: 11,106, 7.9%	Handovers among attending anesthesiologists and handovers among in-room anesthesia providers including residents and fellows, CRNAs, and SRNAs; the number of handovers was defined as a sum of transitions of care during the case timeline within the in-room provider group and within the attending anesthesiologist group	Age, sex, race, ASA physical status, start time of surgery, principal diagnosis and procedure, anesthesia provider breaks modeled as a categorical variable with two levels, duration of surgery	A collapsed composite of in-hospital mortality and six major morbidities (serious cardiac, respiratory, gastrointestinal, urinary, bleeding, and infectious complications based on secondary diagnosis codes in addition to the primary diagnosis): handover OR 0.975 (0.895–1.022); breaks OR 0.933 (0.890–0.977)	Major complications and/or death within 30 days of surgery based on the ACS-NSQIP-defined outcomes (acute renal failure, bleeding that requires transfusion of 4+ packed erythrocytes, cardiac arrest, coma, myocardial infarction, unplanned intubation, prolonged [more than 48 h] ventilation, pneumonia, stroke, wound disruption, surgical site infection, sepsis, and systemic inflammatory response syndrome): handover OR 0.868 (0.718–1.049); breaks OR 0.855 (0.760–0.962)
Jones et al. (16)	One AH: 5,941, 1.9%	The complete intraoperative handover of anesthesia care from one physician anesthesiologist to another physician anesthesiologist	Sex, age, comorbidities with a 5-year look-back window (hypertension, coronary artery disease, congestive heart failure, peripheral vascular disease, diabetes, previous stroke or transient ischemic attack, chronic liver disease, cancer, chronic renal disease, and chronic obstructive pulmonary disease), duration of the surgery (in deciles), years since medical school graduation for the primary anesthesiologist, region of the province, type of hospital (academic or not), whether the surgery was elective or urgent/emergent, and the type of surgery performed	A composite of all-cause death: RR 1.45 (1.19–1.76), readmission to any hospital in the province within 30 days: RR 1.18 (0.98–1.41); major postoperative complications within 30 days of the index surgery: RR 1.25 (1.16–1.34)	Three separate components of the primary outcome: the incidence of postoperative ICU admission RR 1.12 (1.06–1.19); hospital length of stay, the number of emergency department visits in Ontario within 90 days of the index surgery: RR 0.99 (0.92–1.07)
Shah et al. (17)	Attending handoff: 2,586, 21.3%	Handoff at the attending level; a complete handover of care defined as the presence of more than one attending and more than one non Attending	Age, sex, ASA physical status classification, case length, surgical case complexity, evening/weekend start time	A composite outcome of NSQIP postoperative occurrences (progressive or acute renal failure, cardiac arrest requiring cardiopulmonary resuscitation, stroke, any type of surgical site infection or sepsis, myocardial infarction, unplanned intubation, mechanical ventilation greater than 48 h, pneumonia, deep vein thrombosis, venous thromboembolism, urinary tract infection, or readmission within 30 days): attending handoff OR 1.05 (0.92–1.19); complete handoff OR 1.12 (0.96–1.31)	NA
Hannan et al. (18)	One AH: 8,798, 8.5%	Patients with complete anesthesia handovers were defined as patients whose anesthesiologists at the beginning and end of the procedure were different	Surgery duration, surgery start time, numerous demographics, type and priority of cardiac surgery, specific coronary vessels diseased, comorbidities, ventricular function, hemodynamic status, and previous myocardial infarction	In-hospital/30-day mortality OR after IPTW: 1.16 (1.01–1.32)	Major complications during the index admission or within 30 days from the index surgery: OR after IPTW 1.04 (1.01–1.07), readmissions within 30 days of the index admission: OR after IPTW 0.95 (0.89–1.01), and length of stay
Kannampallil et al. (19)	One AH: 5,411, 6.9%	Handover is defined as the change in care between any pair of clinicians responsible for anesthetic care; Handover occurred between pairs of residents, fellows, CRNAs and attending anesthesiologists	Age, sex, race, ASA level, emergent status, duration of surgery, encounter type, major surgery status and surgery start time	A composite of all-cause mortality and major postoperative morbidity within 30 days after surgery OR 0.92 (0.75–1.13)	Cardiac, respiratory, gastrointestinal, urinary, bleeding and infection OR 0.93 (0.75–1.16); all-cause mortality within 30 days: OR 0.8 (0.37–1.73); 30-day hospital readmissions: OR 0.93 (0.75–1.16)
Meersch et al. (11)	One AH: 891, 50.3%	Patients allocated to the handover group were to have at least 1 complete transition of care from one anesthesiologist to another during surgery	Type of surgery, revised cardiac risk index	A 30-day composite of all-cause mortality OR 0.89 (0.72–1.10); hospital readmission: OR 0.80 (0.61–1.05); serious postoperative complications within 30 days after the index surgery: OR 1.02 (0.81–1.28)	19 secondary outcomes including the components of the primary composite, along with ICU and hospital lengths of stay
Sun et al. (20)	One AH: 1,926, 1.9%	Complete handover of anesthesia care, where the case is completed by the replacement anesthesiologist	Not mentioned	Mortality within 30 days: HR 1.50 (1.25–1.81); mortality within 1 year after surgery: HR 1.52 (1.31–1.76)	Patient-defined adverse cardiac and noncardiac events within 30 days: HR 1.13 (0.92–1.39); PACE within 1 year: RR 1.00 (0.85–1.8); ICU stay: RR 1.32 (1.22–1.41), and hospital lengths of stay: RR 1.16 (1.09–1.23)
Bloom et al. (21)	One AH: 1,087, 18.3%; two or more AH: 224, 3.8%	A complete non-transient transition of anesthesia care between attending anesthesiologists		Operative mortality (death during index admission or within 30 days of discharge) OR for single AH: 1.15 (0.79–1.67); OR for multiple AH: 0.83 (0.36–1.90)	Readmission within 30 days of index hospitalization: OR for single AH 1.05 (0.84–1.30); OR for multiple AH 0.86 (0.54–1.36); prolonged hospital length of stay (defined as greater than 14 days) OR for single AH 1.07 (0.84–1.36), OR for multiple AH 1.12 (0.70–1.78); surgical site infection, renal failure, and a composite of major complications occurring during the index hospitalization: OR for single AH 0.95 (0.77–1.16), OR for multiple AH 1.20 (0.82–1.76). The composite of major complications included one or more of the following: cardiac arrest, deep sternal wound infection, deep vein thrombosis, gastrointestinal complications, paralysis, pneumonia, prolonged mechanical ventilation, postoperative reintubation, pulmonary embolism, renal failure, reoperation for bleeding, reoperation for other reasons, sepsis, stroke, and surgical site infection
Saha et al. (22)	One AH: 48,986, 40.4%	The attending anesthesiologist or medically directed individual changed during the anesthetic, defined as anesthesia start to anesthesia end as documented in the electronic medical record. Any change in attending anesthesiologist, without regard to duration, was treated as a handover	Age, sex, race, BMI, Charlson comorbidity index, ASA physical status, weekday day surgery number, duration of anesthesia episode in minutes, GA used in the operation, anesthesia start hour, emergent surgery or elective surgery, et al	A composite of all-cause mortality in the 30 days following surgery and occurrence of postoperative morbidity: OR 1.09 (1.05–1.12)	Morbidity composite: OR 1.09 (1.05–1.13); 30 day all–cause mortality: OR 1.08 (1.04–1.14); one–year all–cause mortality: OR 1.07 (1.02–1.13); safety event (activation of the rapid response team or cardiac arrest team), unplanned ICU admission: OR 1.27 (1.12–1.35); hospital readmission within 30 days after discharge: OR 1.09 (1.04–1.14); remaining intubated at case end: OR 1.04 (0.98–1.14), postoperative reintubation: OR 1.11 (1.05–1.8); length of stay index more than one (indicating actual length of stay higher than predicted length of stay): OR 1.28 (1.24–1.32); postoperative visual or verbal analogue pain score >5/10, and emergency department visit within 30 days after discharge: OR 1.08 (1.03–1.14).
Zhang et al. (23)	One AH: 2,319, 33.3%	A complete handover of intraoperative anesthesia care was defined when the outgoing anesthesiologist transferred patient care to the incoming anesthesiologist and no longer returned	Age, sex, body mass index, and history of smoking and alcohol use; preoperative comorbidities included hypertension, ischemic heart disease, history of heart failure, atrial fibrillation, previous stroke, diabetes mellitus, renal dysfunction, chronic obstructive pulmonary disease, and pre-existing anemia; preoperative medications included antihypertensives, insulin, and oral hypoglycemic agents; general status included American Society of Anesthesiologist (ASA) physical status and revised cardiac risk index; surgery-related data included site, type, and duration of surgery, surgery for cancer; anesthesia-related data included duration of one-lung ventilation, fluid infusion rate, blood transfusion, occurrence of intraoperative hypotension, use of vasopressors, number of concurrent anesthesia care, working experience of senior anesthesiologists, and type of patient-controlled analgesia	A composite of major adverse cardiovascular events (MACEs) including acute myocardial infarction, new onset congestive heart failure, non-fatal cardiac arrest, and cardiac death, that occurred within 7 days after surgery: OR 1.24 (1.03–1.50)	Admission to ICU: OR 1.27 (1.03–1.57); individual component of MACEs within 7 days [acute myocardial infarction: OR 1.24 (1.02–1.52), congestive heart failure: OR 0.87 (0.57–1.32), cardiac death: OR 1.00 (0.14–7.09), ICU admission after surgery: OR 1.27 (1.03–1.57)], length of hospital stay after surgery: OR 0.96 (0.90–1.02); the occurrence of pulmonary complications within 7 days after surgery: OR 1.00 (0.75–1.33).

Periop MI, postoperative myocardial infarction; CVA, cerebral vascular accident; CVVH, continuous veno-veno hemodialysis; Vent >48 hr, mechanical ventilation for longer than 48 h postoperatively.

Six different adverse outcomes were analyzed in these fourteen studies, including composite in-hospital mortality and morbidity (*n* = 8) (3, 10, 11, 15–17, 19, 22), in-hospital mortality (*n* = 10) (10, 11, 13, 16, 18–23), morbidity (*n* = 10) (10, 11, 13, 15, 18–23), ICU stay (n = 3)(15, 22, 23), readmission within 30 days (*n* = 6) (11, 15, 18, 19, 21, 22), the number of emergency department visits within 90 days of the index surgery (*n* = 3) (11, 15, 22).

### Risk of bias

The methodological quality of all included observational studies was independently assessed using NOS. It evaluates study quality across three core domains: participant selection, comparability between study groups, and outcome assessment, with a maximum possible score of nine stars (a score of seven or more stars indicates high methodological quality). In detail, each of the 13 included studies obtained 4 stars in the Selection domain, 2 stars in the Comparability domain, and 2 stars in the Outcome domain, yielding a total score of 8 stars per study, which reflects good overall methodological quality. Nevertheless, all the 13 included studies shared a common methodological limitation in the NOS assessment: clear descriptions related to follow-up (including follow-up duration and completeness) were not explicitly mentioned. With guidance from the instructions of the Cochrane Collaboration, the last RCT was only identified with a high risk of bias in allocation concealment as the supervisors were unblinded when allocating handovers. As fewer than 10 individual studies were included in each analysis, we did not assess publication bias (24).

### Association of anesthesia handovers with patient outcomes

**Figure 2:**
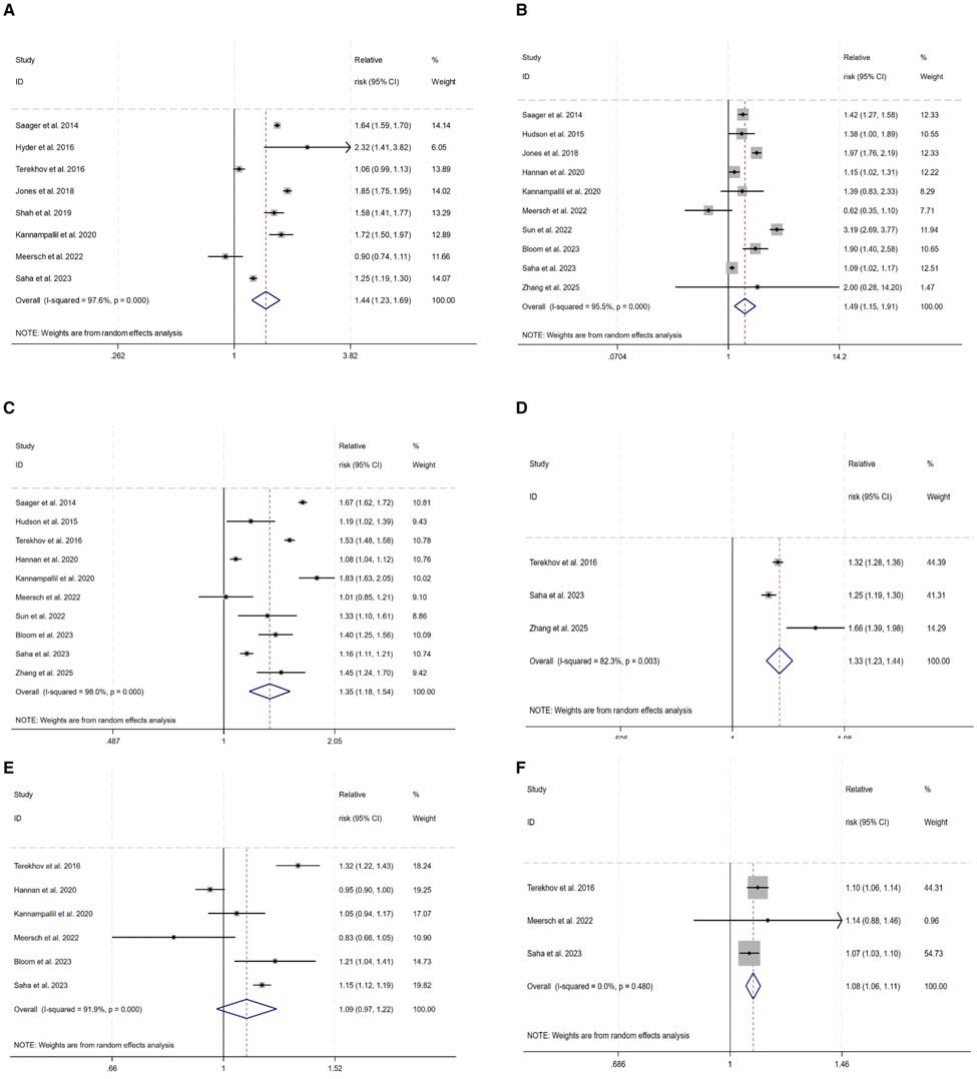
Forest plot of association between anesthesia handovers and pooled aRR of adverse outcomes. **(A)** Forest plot of association between anesthesia handover and pooled aRR of composite in-hospital mortality and morbidity (test of RR = 1: z = 5.10, *P* = 0.000); **(B)** Forest plot of association between anesthesia handover and pooled aRR of in-hospital mortality (test of RR = 1: z = 3.08, *P* = 0.002); **(C)** Forest plot of association between anesthesia handover and pooled aRR of morbidity (test of RR = 1: z = 4.40, *P* = 0.000); **(D)** Forest plot of association between anesthesia handover and pooled aRR of ICU stay (test of RR = 1: z = 7.02, *P* = 0.000); **(E)** Forest plot of association between anesthesia handover and pooled aRR of patients' readmission within 30 days (test of RR = 1: z = 7.02, *P* = 0.000); **(F)** Forest plot of association between anesthesia handover and pooled aRR of the number of emergency department visits within 90 days of the index surgery (test of RR = 1: z = 1.5, *P* = 0.133).

As shown in Figure 2A, eight included studies described the association between anesthesia handover and relative risk of composite in-hospital mortality and morbidity, with a pooled aRR of 1.44 (95% CI 1.23–1.69, *P* < 0.001), with high heterogeneity of I^2^ = 97.6%. Figures 2A–F showed a pooled aRR of 1.49 (95% CI 1.15–1.91, *P* = 0.002, I^2^ = 95.5%) for in-hospital mortality, 1.35 (95% CI 1.18–1.54, *P* < 0.001, I^2^ = 98.0%) for morbidity, 1.33 (95% CI 1.23–1.44, *P* < 0.001, I^2^ = 82.3%) for ICU stay, 1.09 (95% CI 0.97–1.22, *P* = 0.133, I^2^ = 91.9%) for readmission within 30 days, 1.08 (95% CI 1.06–1.11, *P* < 0.001, I^2^ = 0.0%) for the number of emergency department visits within 90 days of the index surgery.

**Figure 3:**
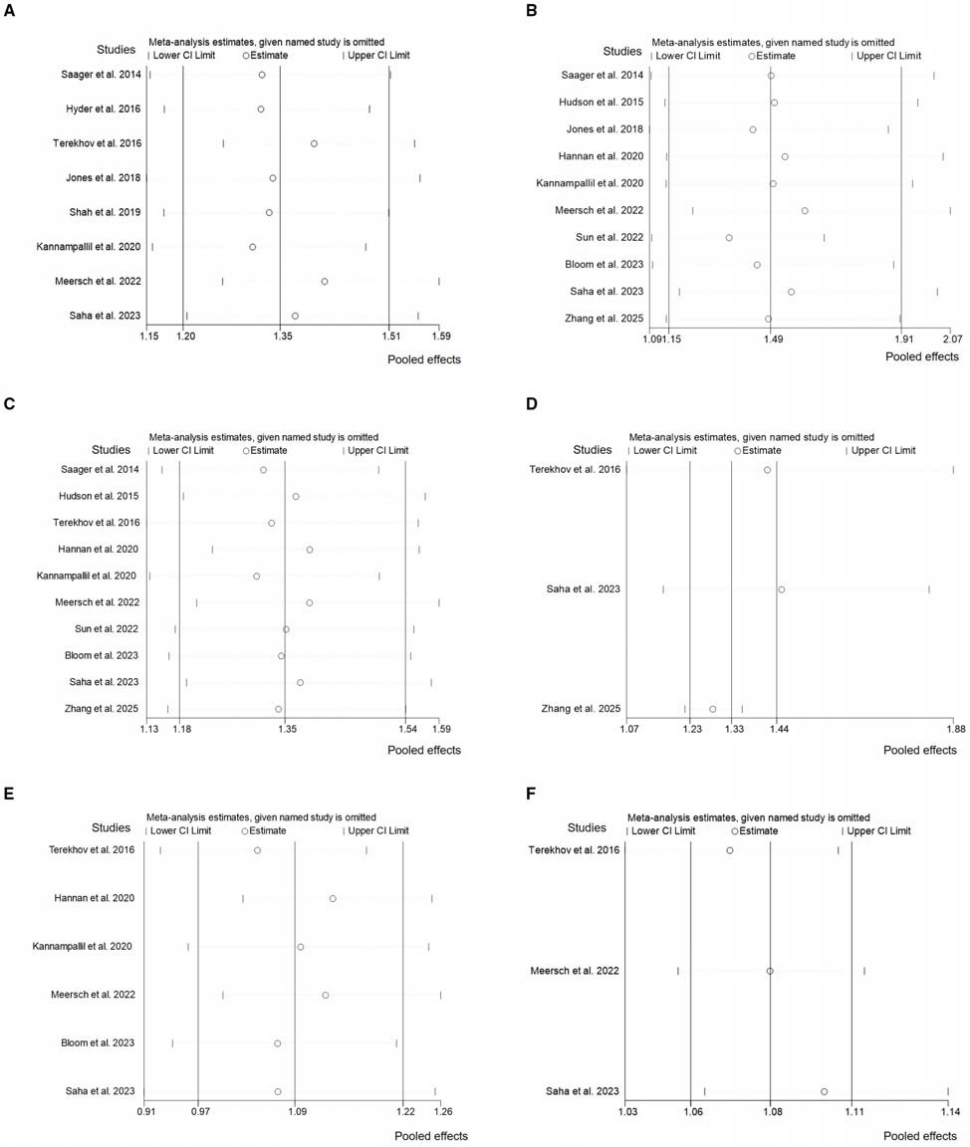
Sensitivity analysis of association between anesthesia handovers and pooled aRR of adverse outcomes. **(A)** Sensitivity analysis of association between anesthesia handover and pooled aRR of composite in-hospital mortality and morbidity; **(B)** Sensitivity analysis of association between anesthesia handover and pooled aRR of in-hospital mortality; **(C)** Sensitivity analysis of association between anesthesia handover and pooled aRR of morbidity; **(D)** Sensitivity analysis of association between anesthesia and pooled aRR of ICU stay; **(E)** Sensitivity analysis of association between anesthesia and pooled aRR of patients' readmission within 30 days; **(F)** Sensitivity analysis of association between anesthesia and pooled aRR of the number of emergency department visits within 90 days of the index surgery.

We conducted leave-one-out sensitivity analysis to determine the stability and robustness of the pooled effect estimates (Figure 3). This demonstrated that the statistically significant association between the composite outcome of in-hospital mortality or morbidity and handover remained robust and unaffected following the sequential exclusion of each individual study included in the meta-analysis.

**Figure 4:**
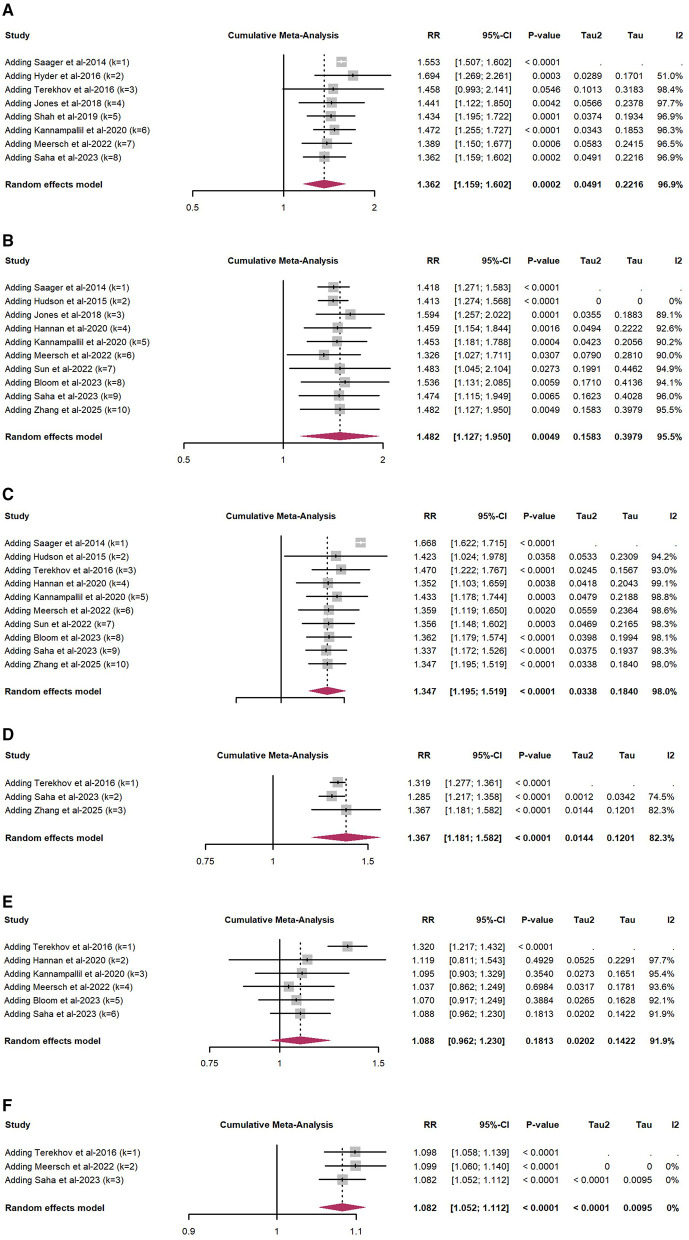
Cumulative meta-analysis of association between anesthesia handovers and pooled aRR of adverse outcomes. **(A)** Cumulative meta-analysis of association between anesthesia handover and pooled aRR of composite in-hospital mortality and morbidity; **(B)** Cumulative meta-analysis of association between anesthesia handover and pooled aRR of in-hospital mortality; **(C)** Cumulative meta-analysis of association between anesthesia handover and pooled aRR of morbidity; **(D)** Cumulative meta-analysis of association between anesthesia and pooled aRR of ICU stay; **(E)** Cumulative meta-analysis of association between anesthesia and pooled aRR of patients' readmission within 30 days; **(F)** Cumulative meta-analysis of association between anesthesia and pooled aRR of the number of emergency department visits within 90 days of the index surgery.

Cumulative meta-analysis was also performed (Figure 4). As studies were gradually incorporated, the combined overall aRRs became increasingly stable despite significant heterogeneity, indicating that the newly added studies did not exert a subversive impact on overall adverse outcomes.

Subgroup analysis indicated that heterogeneity may not originate from the different severities of surgery or handover levels (Supplementary Appendix 3).

## Discussion

This systematic review included 13 retrospective cohort studies and one RCT that evaluated the impact of anesthesia handovers on adverse patient outcomes. The findings revealed a considerable positive correlation between anesthesia handovers and various important patient outcomes, including composite in-hospital mortality and morbidity, in-hospital mortality, morbidity, ICU length of stay, and number of emergency department visits within 90 days of index surgery. In contrast, the relationship between anesthesia handovers and readmission within 30 days was less significant. In addition, the association was not influenced by surgery severity or exact handover levels.

We found that the handover process increased the relative risk of composite in-hospital mortality and morbidity by 44% compared with the no handover process during surgical procedures. In this updated systematic review and meta-analysis, these results are consistent with the existing literature suggesting that anesthesia handovers, as a critical transition point in perioperative care, may introduce communication gaps, information loss, or human error, thereby increasing the likelihood of adverse events (4, 20). Nevertheless, caution is still warranted when concluding that an intraoperative anesthesia handover exerts an adverse impact on the composite outcomes of in-hospital mortality and morbidity among surgical patients. Key considerations justifying this caution include the limited retrospective design of the available evidence base and the ambiguity surrounding the potential causal relationships between these variables. The majority of included studies were observational studies with relatively small sample sizes and single-center design, which may compromise the external validity and generalizability of the pooled estimates. Large-scale multicenter trials are warranted to confirm our findings.

A key challenge in interpreting the results of this meta-analysis is the extremely high heterogeneity observed across most outcomes (I^2^ ranging from 82.3 to 97.6%), which is a common issue in observational meta-analyses investigating perioperative interventions. To address this, we performed subgroup, sensitivity, and cumulative meta-analyses. Leave-one-out sensitivity analysis confirmed the robustness of the pooled results for composite in-hospital mortality and morbidity, as sequential exclusion of any single study did not alter the statistical significance of the association, indicating that no individual study unduly influenced the overall conclusion. Cumulative meta-analysis further demonstrated that the combined effect size became increasingly stable as more studies were incorporated, suggesting that the addition of new research did not reverse the observed association between anesthesia handovers and adverse outcomes.

To reduce the risk of operational errors and medical mistakes caused by frontline work fatigue and sleep deprivation, anesthesia handover is necessary and indispensable. However, reducing working hours may inadvertently increase the frequency of intraoperative anesthesia handovers, a change that comes at the cost of heightened risk of serious adverse complications and adverse events. Frequent handover miscommunication is one of the primary causes of such risks (19). Risk of inadequate information exchange happened more frequently in severer patients (10, 16). In complex clinical environments, the absence of standardized communication checklists, coupled with multitasking, distractions, and time constraints, often leads to information gaps, delayed medical intervention, or oversight of patients' surgical status (19). For example, a well-designed standardized handover checklist primarily comprises eight core modules (patient-related information, surgical details, anesthesia records, leadership coordination, teamwork dynamics, closed-loop communication protocols, the ability to raise critical questions, and workload management)(19). With more than 24 specific items included, it is reasonable to infer that extensive information omissions are likely to occur during verbal handovers. Additionally, recipients often require extra time to identify and familiarize themselves with the key points conveyed by the caregiver. In this context, verbal handovers that fail to prioritize and emphasize critical information ultimately result in a significant wastage of time.

In our study, neither the type of surgical procedure nor the competency level of the anesthesia handover personnel proved to be decisive factors in altering the impact of intraoperative handovers on the occurrence of adverse outcomes. Nevertheless, in real-world clinical settings, a clear trend emerges: as surgical procedures become more complex or urgent, anesthesiologists and nurse anesthetists are increasingly inclined to administer anesthesia without intraoperative handovers. Their primary objective in avoiding intraoperative anesthesia handover whenever feasible is to uphold the highest standards of patient safety throughout the surgical process.

This preference can be traced back to the pivotal role of human factors, which empower anesthesiologists and nurse anesthetists to fulfill their professional responsibilities with precision in emergency or high-stake scenarios. These critical human factors encompass three key dimensions: first, a comprehensive situational awareness of both the patient's physiological condition and the dynamic surgical environment; second, a deep comprehension of the ongoing surgical context, including the specific steps, potential challenges, and surgical team's needs; and third, the ability to proactively anticipate the patient's future clinical trajectory, such as predicting potential hemodynamic changes or anesthesia-related complications.

From this perspective, an intraoperative anesthesia handover inherently carries a significant risk of disrupting situational awareness of the oncoming anesthesiologist (25). Such disruptions often stem from a range of handover-related errors, including inaccuracies in the collection and documentation of key patient information, misinterpretation of shared clinical data (e.g., laboratory results and vital sign trends), delays in translating the acquired information into timely clinical actions, and failure to proactively develop targeted anesthesia management strategies prior to assuming care.

Ultimately, the loss of situational awareness among anesthesiologists, driven by four interrelated factors–inadequate leadership in coordinating the handover process, divided attention due to concurrent clinical demands, ambiguous or incomplete information exchange, and lack of a systemic perspective that integrates patient history, surgical plans, and real-time data—has emerged as a key contributor to the elevated incidence of adverse patient outcomes associated with the intraoperative anesthesia handover process.

Notably, existing clinical evidence has consistently documented inadequate handover communication as a major contributing factor to perioperative adverse events, further underscoring the urgency to address handover-related challenges. Consequently, optimizing anesthesia handover processes should be prioritized as a critical corrective strategy to minimize the harm to surgical patients (26). Key components of this optimization include standardizing the content and format of the handover (e.g., adopting structured frameworks such as SBAR—Situation, Background, Assessment, Recommendation), ensuring that all involved personnel maintain focused attention during information exchange, and establishing clear accountability for the accuracy and completeness of the handover process.

### Strengths and limitations

A key strength of this systematic review is its focus on an extremely common intraoperative clinical scenario that is likely to exert a substantial impact on patient outcomes. Our findings demonstrated a clear association between anesthesia handovers and patient harm, which was consistent with the majority of previous research, with most studies consistently finding a detrimental effect of anesthesia handovers on patient outcomes.

Our study was unable to definitively establish a causal relationship between anesthesia handovers and adverse effects. However, we identified a strong association between these two. Nonetheless, the results of our analysis should be interpreted with caution because of two main limitations of the included evidence: first, all studies were based on observational data and encompassed a wide range of surgical procedure types, which may introduce inherent bias; Second, there were notable inconsistencies in data collection methods, adjustments for confounding factors, and outcome definitions across the studies, coupled with insufficient clarity regarding the specific characteristics of anesthesia handover in each study. Specifically, most studies defined an anesthesia handover solely as a “change in personnel, with minimal to no description of the handover's content, structure, or formatting of critical details that could influence its impact on patient safety.

Substantial heterogeneity was observed across the included studies, underscoring the urgent need for standardized measurement tools, uniform data collection protocols, and rigorous study design in future research. Another limitation is the small number of RCT included in the meta-analysis (only one), which limits the robustness of our conclusions regarding causal inference.

Further research is warranted, as there are limited studies investigating anesthesia handovers and variations in design and methodology. Identifying the specific characteristics of handover processes that influence patient outcomes in different clinical contexts (eg., Surgical specialty, type of anesthesia, elective vs. crisis situation, and academic vs. community center) would be beneficial. Future investigations should also explore the conditions under which handovers either enhance or jeopardize patient safety, as well as determine whether the quantity, quality, or both of handovers significantly influence patient outcomes (26). Once the key aspects of an effective anesthesia handover are established, specific policies and procedures can be developed and implemented.

As the Joint Commission recognizes, handoff standardization can be imperative for enhancing communication quality, particularly since the implementation of a standardized handoff process has been shown to reduce the rate of all medical errors by 23% (27). Jaulin et al. (29) further confirmed that the use of a post-anesthesia team handover checklist reduced hypoxemic events by 5.6 times among adult patients in the post-anesthesia care unit (19). Klocko et al. also recommended structured communication practices to minimize medical errors and improve patient safety during care transitions (28). Taken together, anesthesia handover quality improvement may be helpful in reducing adverse events; however, this requires further investigation.

## Conclusions

Intraoperative anesthesia handover typically elevates morbidity and mortality risks among surgical patients; however, it may also have the potential to enhance patient safety under specific clinical circumstances. To improve the quality of anesthesia handovers, further research is imperative to identify the specific characteristics of handovers that have a meaningful impact on patient safety outcomes.

## Data Availability

The original contributions presented in the study are included in the article/Supplementary material, further inquiries can be directed to the corresponding author.
