# Supplementary material for: Does intraoperative anesthesia handovers associated with adverse outcomes? A systematic review and meta-analysis
Source: Front Med (Lausanne). 2026 Jun 11;13:1815524. doi: 10.3389/fmed.2026.1815524 (PMC13294438; doi:10.3389/fmed.2026.1815524)

**Association between intraoperative anesthesia handoffs and adverse outcome: a systematic review and meta-analysis**

**Supplemental appendix 1.**

**Supplemental appendix table 1-1. Search strategy of PubMed (Search end date: 2025-6-15)**

| # | Searches | Number |
| --- | --- | --- |
| 1 | ((((((((((((((((((Patient handoff[MeSH Terms]) OR (handoff*[Title/Abstract])) OR (hand off*[Title/Abstract])) OR (handover*[Title/Abstract])) OR (hand over*[Title/Abstract])) OR (signout*[Title/Abstract])) OR (sign out*[Title/Abstract])) OR (signover*[Title/Abstract])) OR (sign over*[Title/Abstract])) OR (nursing hand over*[Title/Abstract])) OR (nursing hand off*[Title/Abstract])) OR (nursing handover*[Title/Abstract])) OR (nursing handoff*[Title/Abstract])) OR (intraoperative handoff of care[Title/Abstract])) OR (anesthesia handoff*[Title/Abstract])) OR (anesthesia handover*[Title/Abstract])) OR (intraoperative transitions of anesthetic care[Title/Abstract])) OR (handover of anesthes* care[Title/Abstract])) | 6772 |
| 2 | (((((patient safety[MeSH Terms]) OR (perioperative morbidity[Title/Abstract])) OR (perioperative mortality[Title/Abstract])) OR (adverse event*[Title/Abstract])) OR (postoperative mortality[Title/Abstract])) OR (postoperative morbidity[Title/Abstract]) | 328016 |
| 3 | #1 AND #2 | 966 |
| 4 | ((((meta analysis[MeSH Terms]) OR (meta analysis as topic[MeSH Terms])) OR (meta analysis[Publication Type])) OR (review[Publication Type])) OR (meta-analysis[Title/Abstract]) | 3799209 |
| 5 | #3 NOT #4 | 835 |

**Supplemental appendix table 1-2. Search strategy of Web of Science (Search end date: 2025-6-15)**

| # | Searches | Number |
| --- | --- | --- |
| 1 | (((((((((((((((((TS=(patient handoff)) OR TS=(handoff*)) OR TS=(hand off*)) OR TS=(handover*)) OR TS=(hand over*)) OR TS=(signout*)) OR TS=(sign out*)) OR TS=(signover*)) OR TS=(sign over*)) OR TS=(nursing hand over*)) OR TS=(nursing hand off*)) OR TS=(nursing handover*)) OR TS=(nursing handoff*)) OR TS=(intraoperative handoff of care)) OR TS=(anesthesia handoff*)) OR TS=(anesthesia handover*)) OR TS=(intraoperative transitions of anesthetic care)) OR TS=(handover of anesthes* care) | 937016 |
| 2 | (((((TS=(patient safety)) OR TS=(perioperative morbidity)) OR TS=(perioperative mortality)) OR TS=(adverse event*)) OR TS=(postoperative mortality)) OR TS=(postoperative morbidity) | 1458934 |
| 3 | #1 AND #2 | 40981 |
| 4 | ((TS=(meta*analysis)) OR TS=(meta analysis as topic)) OR TS=(review) | 5875560 |
| 5 | #3 NOT #4 | 31966 |

**Supplemental appendix table 1-3. Search strategy of Embase. (Search end date: 2025-6-15)**

| # | Searches | Number |
| --- | --- | --- |
| 1 | **'clinical handover'**/exp OR **'clinical handover'** | 12876 |
| 2 | 'patient handoff':ti,ab,kw OR 'handoff*':ti,ab,kw OR 'hand off*':ti,ab,kw OR 'handover*':ti,ab,kw OR 'hand over*':ti,ab,kw OR 'signout*':ti,ab,kw OR 'sign out*':ti,ab,kw OR 'signover*':ti,ab,kw OR 'sign over*':ti,ab,kw OR 'nursing hand over*':ti,ab,kw OR 'nursing hand off*':ti,ab,kw OR 'nursing handover*':ti,ab,kw OR 'nursing handoff*':ti,ab,kw OR 'intraoperative handoff of care':ti,ab,kw OR 'anesthesia handoff*':ti,ab,kw OR 'anesthesia handover*':ti,ab,kw OR 'intraoperative transitions of anesthetic care':ti,ab,kw OR 'handover of anesthes* care':ti,ab,kw | 11949 |
| 3 | #1 OR #2 | 22473 |
| 4 | 'patient safety'/exp OR 'patient safety' | 227034 |
| 5 | 'patient safety':ti,ab,kw OR 'perioperative morbidity':ti,ab,kw OR 'perioperative mortality':ti,ab,kw OR 'adverse event*':ti,ab,kw OR 'postoperative mortality':ti,ab,kw OR 'postoperative morbidity':ti,ab,kw | 614327 |
| 6 | #4 OR #5 | 756102 |
| 7 | #3 AND #6 | 3684 |
| 8 |  |  |
| 9 | 'meta analysis'/exp OR 'meta analysis' OR (meta AND ('analysis'/exp OR analysis)) OR 'meta analysis':it OR review:it | 3799072 |
| 10 | #8 NOT #9 | 3349 |

**Supplemental appendix table 1-4. Search strategy of Cochrane library trials. (Search end date: 2025-6-15)**

| # | Searches | Number |
| --- | --- | --- |
| 1 | MeSH descriptor:[patient handoff] explode all trees | 66 |
| 2 | (patient handoff):ti,ab,kw OR (handoff*):ti,ab,kw OR (hand off*):ti,ab,kw OR(handover*):ti,ab,kw OR (hand over*):ti,ab,kw OR (handoff*):ti,ab,kw OR (signout*):ti,ab,kw OR (sign out*):ti,ab,kw OR (signover*):ti,ab,kw OR (sign over*):ti,ab,kw OR (nursing hand over*):ti,ab,kw OR (nursing hand off*):ti,ab,kw OR (nursing handover*):ti,ab,kw OR (nursing handoff*):ti,ab,kw OR (intraoperative handoff of care):ti,ab,kw OR (anesthesia handoff*):ti,ab,kw OR (anesthesia handover*):ti,ab,kw OR (intraoperative transitions of anesthetic care):ti,ab,kw OR (handover of anesthes*):ti,ab,kw | 49112 |
| 3 | #1 OR #2 | 49112 |
| 4 | MeSH descriptor:[Patient safety] explode all trees | 1042 |
| 5 | (patient safety):ti,ab,kw OR (perioperative morbidity):ti,ab,kw OR (perioperative mortality):ti,ab,kw OR (adverse event*):ti,ab,kw OR (postoperative mortality):ti,ab,kw OR (postoperative morbidity):ti,ab,kw | 268911 |
| 6 | #4 OR #5 | 268911 |
| 7 | #3 AND #6 | 9910 |
| 8 | Filter 1: Trials | 9746 |

**Supplemental appendix table 1- 5. Search strategy of MEDLINE. (Search end date: 2025-6-15)**

| # | Searches | Number |
| --- | --- | --- |
| 1 | patient handoff.sh. | 1752 |
| 2 | (patient handoff or hand off* or patient handover or hand over or signout* or sign out* or sign over* or signover or nursing hand off* or nursing hand over* or nursing handoff* or nursing handover* or intraoperative handoff of care or anesthesia handoff* or anesthesia handover* or intraoperative transitions of anesthetic care or handover of anesthes*).mp. [mp=title, book title, abstract, original title, name of substance word, subject heading word, floating sub-heading word, keyword heading word, organism supplementary concept word, protocol supplementary concept word, rare disease supplementary concept word, unique identifier, synonyms, population supplementary concept word, anatomy supplementary concept word] | 4150 |
| 3 | #1 OR #2 | 4150 |
| 4 | Patient safety.sh. | 27555 |
| 5 | (patient safety or perioperative morbidity or perioperative mortality or adverse event* or postoperative mortality or postoperative morbidity).mp. [mp=title, book title, abstract, original title, name of substance word, subject heading word, floating sub-heading word, keyword heading word, organism supplementary concept word, protocol supplementary concept word, rare disease supplementary concept word, unique identifier, synonyms, population supplementary concept word, anatomy supplementary concept word] | 363621 |
| 6 | #4 OR #5 | 363621 |
| 7 | #3 AND #6 | 1034 |
| 8 | meta analysis.pt. or meta analysis.ti. or meta analysis.ab. or meta$analysis.kw. or review.kw. | 352305 |
| 9 | #7 NOT #8 | 1023 |

**Supplemental appendix 2. Evaluation of the Newcastle-Ottawa scale for quality of 13 included studies.**

| No. | **Research** | **A** | **B** | **C** | **D** | **E** | **F** | **G** | **H** | **I** | **Total score** | **Quality grade** |
| --- | --- | --- | --- | --- | --- | --- | --- | --- | --- | --- | --- | --- |
| 1 | Saager et al. 2014 | 1 | 1 | 1 | 1 | 1 | 1 | 1 | 1 | 0 | 8 | Good |
| 2 | Hudson et al. 2015 | 1 | 1 | 1 | 1 | 1 | 1 | 1 | 1 | 0 | 8 | Good |
| 3 | Anastasian et al. 2016 | 1 | 1 | 1 | 1 | 1 | 1 | 1 | 1 | 0 | 8 | Good |
| 4 | Hyder et al. 2016 | 1 | 1 | 1 | 1 | 1 | 1 | 1 | 1 | 0 | 8 | Fair |
| 5 | Terekhov et al. 2016 | 1 | 1 | 1 | 1 | 1 | 1 | 1 | 1 | 0 | 8 | Good |
| 6 | Jones et al. 2018 | 1 | 1 | 1 | 1 | 1 | 1 | 1 | 1 | 0 | 8 | Good |
| 7 | Shah et al. 2019 | 1 | 1 | 1 | 1 | 1 | 1 | 1 | 1 | 0 | 8 | Good |
| 8 | Hannan et al. 2020 | 1 | 1 | 1 | 1 | 1 | 1 | 1 | 1 | 0 | 8 | Good |
| 9 | Kannampallil et al. 2020 | 1 | 1 | 1 | 1 | 1 | 1 | 1 | 1 | 0 | 8 | Good |
| 10 | Sun et al. 2022 | 1 | 1 | 1 | 1 | 1 | 1 | 1 | 1 | 0 | 8 | Good |
| 11 | Bloom et al. 2023 | 1 | 1 | 1 | 1 | 1 | 1 | 1 | 1 | 0 | 8 | Good |
| 12 | Saha et al. 2023 | 1 | 1 | 1 | 1 | 1 | 1 | 1 | 1 | 0 | 8 | Good |
| 13 | Zhang et al. 2025 | 1 | 1 | 1 | 1 | 1 | 1 | 1 | 1 | 0 | 8 | Good |

Note: A, Representativeness of the exposed cohort; B, Selection of the non-exposed cohort; C, Ascertainment of exposure; D, Demonstration that the outcome (dementia/cognitive impairment) was not present at the start of the study; E, Comparability of cohorts according to the design and analysis; F, The study controls for additional factors (age, sex, ASA physical status, surgery type, emergency or elective surgery, etc.); G, Assessment of adverse outcome using validated assessment tools; H, Follow-up was long enough for the outcomes to occur; I, Adequacy of follow-up of cohorts. Maximum possible score=9.

**Supplemental appendix 3.** **Subgroup analysis of association between anesthesia handovers and pooled aRR of adverse outcomes.**

Supplemental appendix 3-1. subgroup analysis of association between anesthesia handover and relative risk of composite in-hospital mortality and morbidity.

Supplemental appendix 3-2. Subgroup analysis of association between anesthesia handover and pooled aRR of in-hospital mortality.
Supplemental appendix 3-3. Subgroup analysis of association between anesthesia handover and pooled aRR of morbidity.
Supplemental appendix 3-4. Subgroup analysis of association between anesthesia and ICU stay.
Supplemental appendix 3-5. Subgroup analysis of association between anesthesia and pooled aRR of patients’ readmission within 30 days.
Supplemental appendix 3-6. Subgroup analysis of association between anesthesia and pooled aRR of the number of emergency department visits within 90 days of the index surgery.

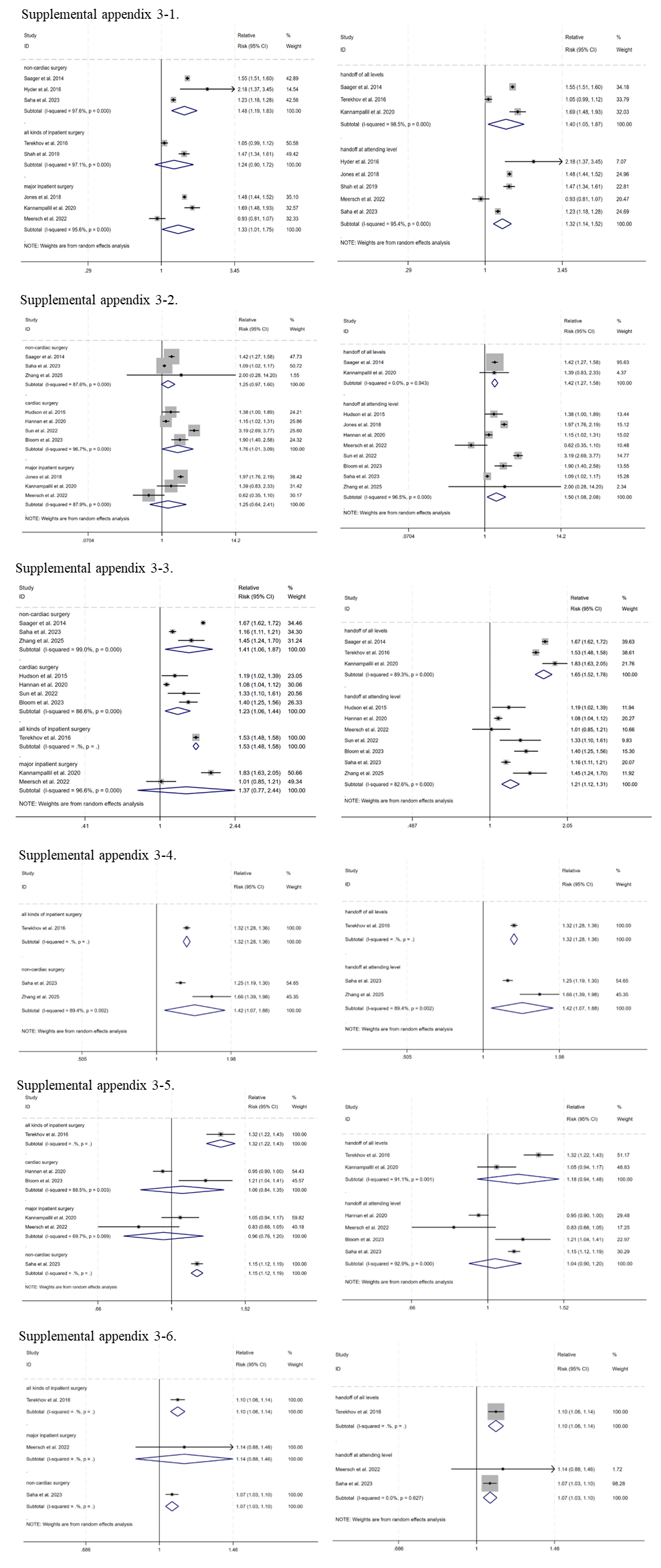

Supplement: Supplementary file 1 [file Supplementary_File_1.docx]
